# Trace Elements in Soil and Urban Groundwater in an Area Impacted by Metallurgical Activity: Health Risk Assessment in the Historical Barga Municipality (Tuscany, Italy)

**DOI:** 10.3390/ijerph192013419

**Published:** 2022-10-17

**Authors:** Riccardo Petrini, Lisa Ghezzi, Simone Arrighi, Lisa Genovesi, Chiara Frassi, Luca Pandolfi

**Affiliations:** Department of Earth Sciences, University of Pisa, Via S. Maria 53, 56126 Pisa, Italy

**Keywords:** trace elements, health risk assessment, heavy metals, Tuscany (Italy), soil contamination, urban environment

## Abstract

Trace elements were measured in soil and groundwater collected within the Fornaci di Barga urban area (Serchio River Valley, Tuscany, Italy), a territory that integrates natural assets with touristic vocation, impacted by long-lasting metallurgical activity. Epidemiological studies highlighted that the area surrounding the industrial plants is characterized by a persistent excess of diseases, attributed to heavy metal pollution. Soils were taken in school gardens, public parks, sport grounds and roadsides. The results indicate that Cu, Zn and Cd represent the main contaminants in surface soil, likely originated by deposition of airborne particulate matter from metallurgical activity. Risk assessment considering soil ingestion and dermal contact exposure routes revealed that the cadmium Hazard Quotient approaches unity for children, and the cadmium risk-based concentration obtained by combining exposure information with toxicity data is only slightly lower compared with the cadmium maximum concentration actually measured in soil. Groundwater does not show evidence of trace metal contamination, suggesting that the migration of contaminants from soil to subsurface is a slow process. However, assessment of the possible interconnections between shallow and deep-seated aquifers requires monitoring to be continued. The obtained results highlight the possible link between space clusters of diseases and metal concentration in soil.

## 1. Introduction

The quantitative assessment of environmental soil quality in urban green spaces is a priority to enhance resilience and to promote high standard of living [1]. Soils in metropolitan areas are variably affected by human activities [2,3,4]; indeed, urban soils contain different materials related to the urban development distributed in anthropogenic layers and act as sinks for pollutants from localized and/or diffused manifold anthropogenic sources. These include automotive traffic, industry, fossil fuel combustion, emissions from municipal incinerators and waste disposal [5]. The urban soil structure ranges from relatively undisturbed to completely man-made, lacking the established horizons that characterize natural soil [6] and influencing the retention and transport of contaminants through the subsurface. Potentially toxic elements in soils have different mobility and can be transported by rainwater during runoff and/or leached from soil by infiltrating water [7], possibly extending the pollution out of the urban area. Once soil is contaminated by PTE such as heavy metals it is difficult to recover, yielding accumulation gradients and long-term effects along urbanization [8,9]. The prolonged exposure to contaminated soil represents a potential hazard for inhabitants, due to both direct pathways that include dermal contact, ingestion and dust inhalation [10], and through indirect routes of exposure such as the food chain [11]. Potentially toxic elements in urban soils may exert chronic effects of toxicity, especially on preschool children that have the tendency to place objects and their fingers in their mouths, unintentionally eating soil while spending their time in urban parks and kindergartens [12,13]. In Italy, the Code of Environment (Legislative Decree 152/2006 which regroups in a single legislative text the environmental laws previously contained in several decrees) recognizes the importance of soil in public green spaces and for residential use, posing restrictive concentration thresholds for PTE and health risk models and tools, to estimate the probability of adverse health effects in humans exposed.

The present study aims at depicting the distribution of PTE in soil and groundwater in the urban setting of Fornaci di Barga (Lucca), within the historical Barga municipality (Middle Serchio River Valley, Tuscany, Italy). In this area, metallurgical activities situated directly adjacent the residential area of Fornaci di Barga have lasted more than 100 years, creating a potential ecotoxicological hazard by PTE primarily delivered to soil through industrial dust varying in age of settlement. Indeed, health statistics and epidemiological evidence highlighted the prevalence of lung cancer and chronic kidney and cardiovascular disease in the Middle Valley [14,15], with a significantly higher incidence compared with the average of Tuscany. In particular, the incidence of kidney diseases increases approaching the site of current and historical metallurgical manufacturing, as revealed by an ongoing cohort study, and the cause has been postulated as due to environmental pollution by PTE, particularly cadmium [15]. Fornaci di Barga has been included among five pilot cities in Europe for industrial pollution and health impact [16] (CitieS-Health project: https://citieshealth.eu/ (accessed on 1 June 2022)).

The results of this study provide valuable insights to policy makers and urban planners into how to mitigate potential health risks in industrial threatened urban areas.

## 2. Environmental and Geological Setting

**Figure 1:**
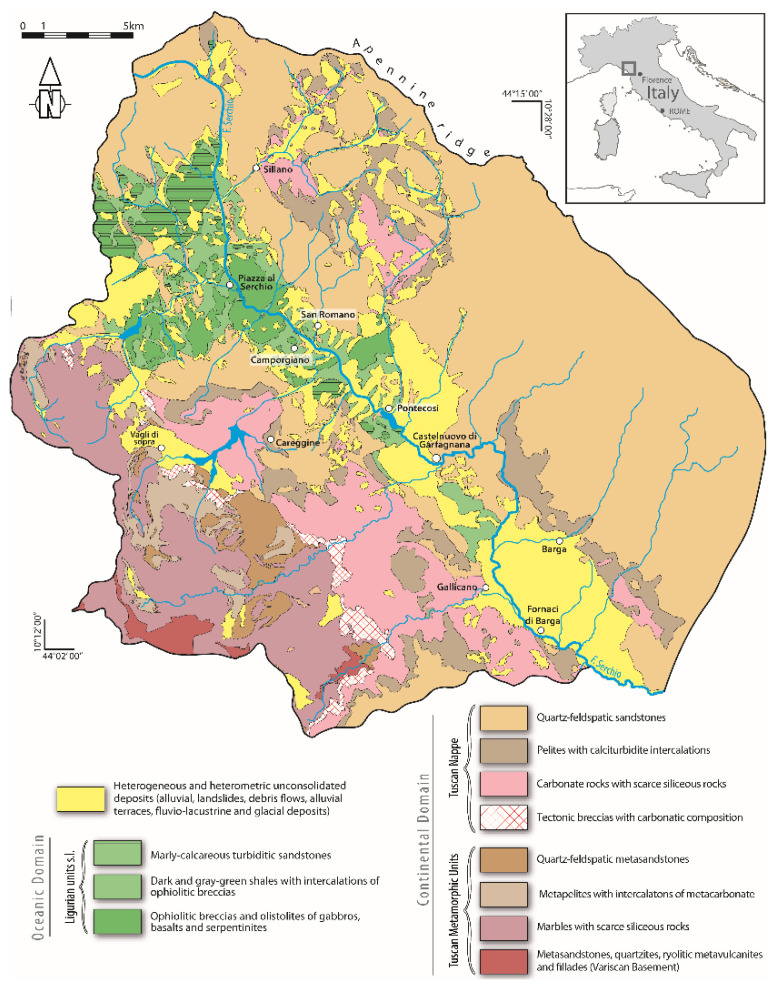
Lithological map of the northern hydrographic basin of Serchio River. The location of the study area, Fornaci di Barga, is shown.

The Fornaci di Barga village belongs to the Barga municipality (Province of Lucca, Tuscany, Italy) in the Serchio River Middle Valley (Figure 1) between the Apuan Alps mountain range and Apennine chain, a territory with cultural and historical heritage, naturalistic assets, and a great touristic vocation. From a geological point of view, the Serchio River Valley is a NW-SE-trending tectonic depression dissecting the architecture of the Neogenic Northern Apennine orogenic belt made up of tectonic units derived from both oceanic (Ligurian Units, composed by ophiolitic rocks and their sedimentary cover) and continental domains (Tuscan Nappe and Tuscan metamorphic Units) (Figure 1). The metamorphic and the non-metamorphic Tuscan units are separated by thick carbonate cataclasites mainly originated during the nappe stacking.

Whatever is the trigger enhancing the exhumation of the Tuscan metamorphic rocks currently exposed in the Apuan Alps [17,18], the tectonic nappe stack of Northern Apennines was affected by an extensional tectonic regime at earlier than 11 Ma [19] with the activation of low- and subsequently of NW-SE trending high-angle faults [20]. The latter are responsible for the last uplift stages of the Apuan Alps region (2–6 Ma) [19] and the development of the tectonic depression of the Serchio River Valley.

The depression is bounded by high-angle normal faults dipping toward east on the west side and toward west on the east side of the Valley. Their activity allows the exposure of the Ligurian units s.l. (i.e., the units at the top of the Apennine nappe stack) at the bottom of the Valley (Figure 1). It also strongly controls the landscape, which shows landforms typical of tectonically active regions with high rates of uplift producing high relief topography with steep mountain slopes (i.e., the Apuan Alps). From early Pliocene to early Pleistocene the tectonic depression was filled by 80 to 320 m-thick continental sequence deposited in fluvial environment associated to debris flows sourced from both sides of the tectonic depression in rapid uplift [21,22,23]. Currently, the best outcrops of these deposits are in the areas of Barga-Fornaci di Barga and Castelnuovo di Garfagnana (Figure 1). These deposits consist of a basal sequence of sands and silty sands interbedded by unsorted to poorly sorted medium to very coarse-grained conglomerates, 20–40 cm thick organic-rich lenses and paleosols [22]. Clasts of conglomerates are mainly represented by Tuscan non-metamorphic lithotypes with a 15–30% of clasts deriving from the Tuscan metamorphic rocks [21]. Clasts are mostly sandstones referable to the youngest formation of the Tuscan Nappe currently exposed in the Apennine main ridge. Moving upward, the sequence is characterized by a dominance of poorly- to well-sorted, medium to coarse-grained and mainly clast-supported conglomerates with interbedded sandy material. Clasts mainly consist of carbonate lithotypes equally derived from metamorphic and non-metamorphic Tuscan units whereas sandstones clasts are subordinates [21].

During middle to late Pleistocene, these sediments were cut by the Serchio River and its tributaries forming different orders of alluvial terraces [22].

Fornaci di Barga develops on one of these fluvial terraces. In the area the thickness of these sediments commonly ranges from 5 to 30 m. From a hydrogeological point of view these deposits have a medium-high to high permeability associated with their primary porosity. They lie on poorly permeable or almost impermeable deposits, formed by Pleistocene clays and sandy clays, which form the bedrock of the unconfined aquifer.

Metallurgical activity in the area started in 1915, and Fornaci di Barga is now one of the three main industrial sites of KME (Engineering Copper Solutions) one of the largest manufacturers in the world of copper and copper alloy preliminary and semi-finished product. Copper represents the second most used non-ferrous metal in industry. Excesses above the regional average of cardiovascular diseases, chronic kidney diseases and malignant tumors such as lung cancer have been reported in the area. A strong tendency to associate environmental pollution with the perceived health profile by inhabitants has been demonstrated [15].

## 3. Materials and Methods

### 3.1. Sampling

**Figure 2:**
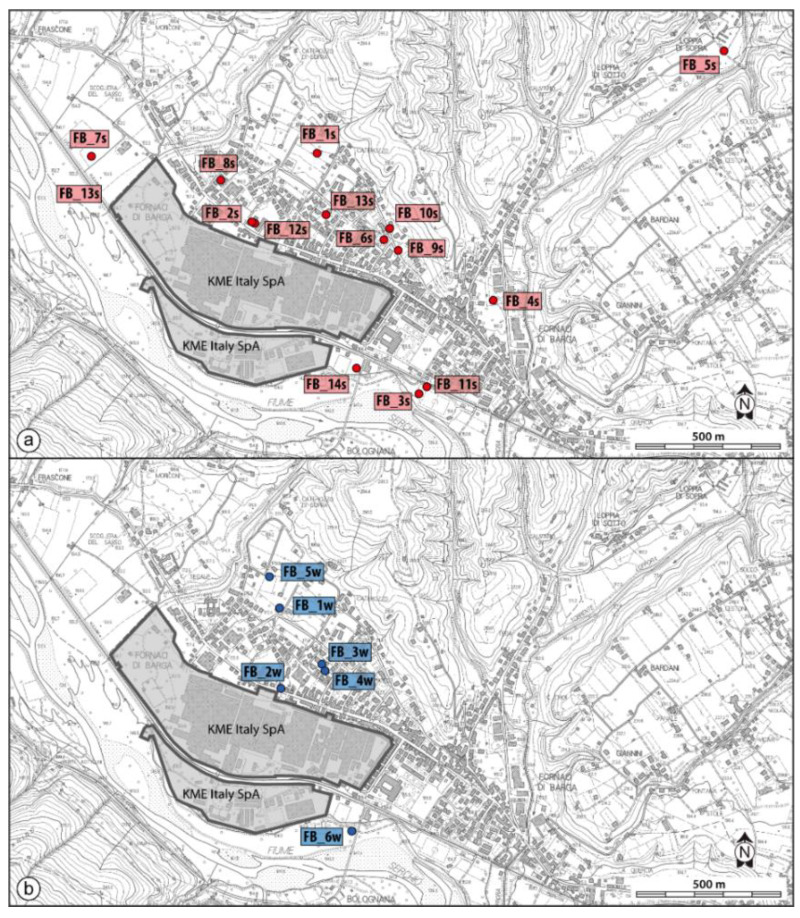
Location of soil (**a**) and groundwater (**b**) sampling stations. Groundwater was sampled in the unconfined aquifer of Fornaci di Barga on July, 2020 through five wells tapping groundwater bodies at different depth (FB_1W, depth 50 m; FB_2W, depth 8 m; FB_3W, depth 7 m; FB_4W, depth 18 m; FB_5W, depth 21 m) and in a piezometer nearby the station FB_14S, using a bailer at depth of 3.5 and 8.7 m below ground level (samples FB_6Wa and FB_6Wb, respectively). The georeferenced distribution of groundwater sampling stations is shown in Figure 2b. Water samples were filtered in the field through 0.45 μm filters and refrigerated in pre-cleaned polyethylene bottles. Ultrapure nitric acid was used as a preservative for major cation and trace element analysis.

Soils were collected in 14 stations during surveys between February and June 2020 (samples FB_1S to FB_14S). Sampling was adapted to the Fornaci di Barga urban pattern, considering different urban land uses including school gardens (FB_1S, FB_2S, FB_9S, FB_12S); sports grounds (FB_3S, FB_11S); private gardens (FB_8S, FB_13S); roadsides (FB_4S, FB_7S), public parks (FB_6S, FB_10S) and riverside fishing access (FB_14S) near KME. One station (FB_5S) was in a peri-urban area. The georeferenced distribution of sampling sites is shown in Figure 2a. Most soils were taken at 0–10 cm depth and in some cases at greater depth (Table 1) by using a hand auger. To quantify the geogenic fingerprint of trace elements in Serchio River Valley sediments, at the watershed scale of interest, fluvial sediments were collected within the reservoir system of the hydroelectric lake of Pontecosi, about 11 km upstream Fornaci di Barga (Figure 1), where the impact of anthropogenic activities is absent. In the lake, sediments were collected in 15 sampling stations during September 2019, both in subaerial domains, to have an archive that includes environmental conditions closed to exchanges with the free water column, using a manual auger (Matest manufacturer, Treviolo, Italy) (composite samples PC1 to PC9 combining all the cores from top up to 1 m depth) and dredged from subaquatic lake floor using a Van Veen grab sampler (samples PC10 to PC15). To compare PTE concentration against Italian guidelines soil and sediment samples were quartered and sieved to separate the <2 mm size fraction for multi-element chemical analysis.

### 3.2. Analytical Procedures

For all instrumental analysis calibration curves were performed prior samples analysis. The minimal number of standard calibration solutions was four and correlation coefficients for calibration curves were better than 0.99, otherwise the calibration curve was repeated. Quality control samples were analyzed one in every ten samples. Quality Control samples included blanks (to control the purity of reagents, the other possible contamination in all work procedures and the memory effect) and check solutions (or check solid matrices) whose elements concentration is known. Certified reference materials (see below) were used to evaluate accuracy and precision of the method.

Soil samples were digested by using the Ethos Easy microwave platform (Milestone Srl, Sorisole, Italy) (US EPA Method 3052, reversed aqua regia). The concentration of a set of trace elements was determined by ICP-MS using the NexION 300X (Perkin Elmer Inc., Waltham, MA, USA). The analytical uncertainty was evaluated by the analysis of soil reference material NIST SRM 2711a (Montana soil). RSD was 5% for Li, Be, Mn, Ni, Ag, Sn, Cd, Tl, Pb, Fe and As, and 10% for Co, Cu, Zn, Sr, Sb, Ba, Th, U, V, and Cr. In general, the accuracy was better than 10%. Soil pH was also measured (ISO 10390). The total mercury content was determined (US EPA Method 7473) by the DMA-80 (Milestone Srl, Sorisole, Italy). NIST 2711a, ERM-CC018 (sandy soil) and MESS-3 (marine sediment) were used as reference materials. RSD was 5% and accuracy was within 10%. Leaching tests on soil samples were performed following the procedure for granular waste EN 12457-2:2002. Briefly, one stage batch test under stirring for 24 h at a liquid to solid ratio of 10 L/kg with particle size below 4 mm and deionized water as liquid was applied.

Water temperature, pH, electrical conductivity (EC), redox potential (Eh), and dissolved oxygen (DO) were measured in the field. Alkalinity (expressed as HCO_3_^−^) was also determined in the field by acidimetric titration. Major ions were determined by ion chromatography using the ICS 900 (Thermo Fischer Scientific, Waltham, MA, USA). RSD was less than 5%. Trace elements in waters and leachates were determined by ICP-MS using a PerkinElmer NexION 300X. Deviations from the certified values of water standards NIST SRM 1640a (trace elements in natural water) and 1643f (trace elements in water) (20 replicates) were less than 5%, except for Cu, Fe, Li and Zn (5–10%). RSD was 10% except for Sr, Mo, Ag, Cd, Sb, Tl, Pb, U and As (5%).

### 3.3. Risk Assessment

Risk analysis was performed following the approaches described both by ASTM standards [24] and United States Environmental Protection Agency (US EPA) guidelines [25] and using the Risk-net software (version 3.1.1pro, September 2019. http://www.reconnet.net/Software.htm (accessed on 1 June 2022)).

The selected exposure routes were surface soil ingestion and dermal absorption in unpaved outdoor areas of residential zones. The soil dust inhalation was not included in risk analysis due to the lack of site-specific data (e.g., ambient air velocity in the mixing zone); it has to be mentioned that inhalation pathways can result in much higher exposure than dermal exposure in many circumstances depending on the size distribution of the dust. In addition, fugitive particulate emission reflects the occurrence of different categories of open dust sources, including re-entrained dust due to traffic traveling over the paved roadway, that would be necessary to estimate a realistic risk assessment through inhalation exposure pathway. Human receptors have been identified with both adults and children. The Hazard Quotient (HQ) for non-carcinogenic chronic (long term) effects in humans was calculated for each exposure pathway [26]. In case of direct ingestion and dermal contact, HQ (i.e HQ_ingestion_ and HQ_derma_l) was calculated by dividing the chronic daily intake (CDI, mg/kg/day) by the corresponding reference dose (RfD, mg/kg/day) [27,28], defined as the maximum daily exposure to a toxic agent that would not produce any appreciable deleterious effects on human health:

(1) $$\mathrm{HQ}=\frac{\mathrm{CDI}}{\mathrm{RfD}}$$

where CDI represents the exposure to a toxic agent, averaged over a long period of time, through ingestion (CDI_ing_) or dermal contact (CDI_derm_), given by:

(2) $${\mathrm{CDI}}_{\mathrm{ing}}=C_{\mathrm{POE}}*\frac{R_{\mathrm{ing}}*\mathrm{EF}*\mathrm{ED}}{\mathrm{BW}*\mathrm{AT}}*10^{-6}$$

(3) $${\mathrm{CDI}}_{\mathrm{derm}}=C_{\mathrm{POE}}*\frac{\mathrm{SA}*\mathrm{SAF}*\mathrm{ABS}*\mathrm{EF}*\mathrm{ED}}{\mathrm{BW}*\mathrm{AT}}*10^{-6}$$

where C_POE_ is the exposure point concentration of contaminant in soil (mg/kg), equal to the concentration at the source (Cs) for direct exposure pathways. For the remaining parameters, recommend values were used [29,30,31]: R_ing_ is the ingestion rate (100 mg/day for adult, 200 mg/day for children); EF is the exposure frequency (350 day/year, maximum value suitable for a residential setting), ED is the exposure duration (24 years for adults, 6 years for children); SA is the exposed skin area (5700 cm^2^ for adults, 2800 cm^2^ for children); SAF is the skin adherence factor (0.07 mg/cm^2^ day for adults, 0.2 mg/cm^2^ day for children); ABS is the dermal absorption factor (chemical specific, unitless); BW is the average body weight (70 kg for adults,15 kg for children); AT is the average time of exposure to non-carcinogens (ED × 365 day/year).

The total risk for a single substance defines a screening level individual Hazard Index (HI_i_), such as:

(4) $${\mathrm{HI}}_{i}={\mathrm{HQ}}_{\mathrm{ingestion}}+{\mathrm{HQ}}_{\mathrm{dermal}}$$

A HI_i_ value less than unity indicates that risk is acceptable [30]. Hazard Index resulting from simultaneous exposure to n non-carcinogens (HI_cum_), is given by:

(5) $${\mathrm{HI}}_{\mathrm{cum}}=\sum_{i=1}^{n}{\mathrm{HI}}_{i}$$

The maximum allowed concentration of contaminants in soil, intended to be protective of human health (soil screening levels–SSLs, according to US EPA guidelines), was obtained by following the Risk Based Corrective Action procedure [24,25]. In this approach, the exposure equations and pathway models are run in reverse to back-calculate the acceptable level of a contaminant concentration in soil corresponding to the target risk. Risk based SSLs for the different outdoor exposure pathways and for residential setting were derived from standardized sets of equations that are based on the updated U.S. Environmental Protection Agency’s human health risk assessment methods [32]. Furthermore, individual SSLs were reduced iteratively dividing each SSL by a corrective factor (f) until an acceptable cumulative risk was obtained. The reduced SSL (SSL/f), ensuring the respect of both the individual and cumulative risks for each contaminant, represents the cumulative soil screening level (SSL_cum_) to be compared with the measured concentrations in soil.

## 4. Results

### 4.1. Soil and Lake Sediments

**Figure 3:**
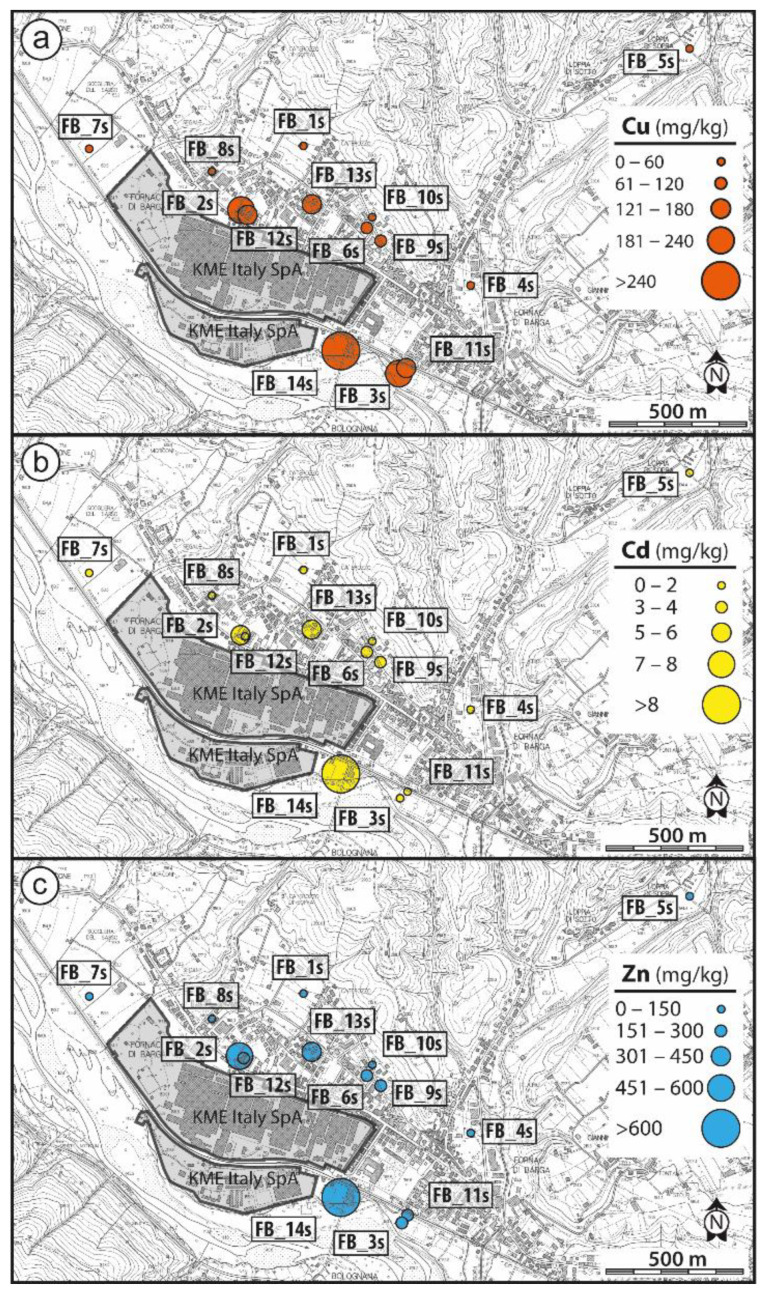
Copper (**a**), Zn (**b**), Cd (**c**) concentration in Fornaci di Barga soil.

Soils represent natural soil reworked artificially; unattached pieces of millimetric to centimetric well rounded or more angular rock fragments are dispersed in a loamy sand matrix. Soil pH is in the range between 4 and 5. Trace elements concentration in Fornaci di Barga soils, including PTE, are given in Table 1. It is observed that in most stations Cu, Zn and Cd exceed the maximum concentration level (MCL) for residential soil use imposed by Italian regulations. In particular, the Ni and Cr content range from 37 to 265 mg/kg and from 55 to 146 mg/kg, respectively; Cu, Zn and Pb range from 35 to 5275 mg/kg, 62 to 27405 mg/kg and 10.6 to 221 mg/kg, respectively; Cd and Sb range from 0.28 mg/kg to 15.8 mg/kg and from 0.20 to 11.7 mg/kg, respectively; As and Hg are in the range from 3.0 mg/kg to 24.5 mg/kg and 0.04 to 2.20 mg/kg, respectively. The areal distribution of Cu, Zn and Cd concentration in soil is shown in Figure 3. Exceedingly high concentrations of pollutants characterize soil in station FB_14S, near the industrial site, suggesting a point source of pollution. The data indicate that most of the collected soils are being polluted at least for one potentially toxic element.

To assess the potential for solubility and release of inorganic pollutant from soil to groundwater, leaching tests were performed on FB_13S and FB_14S soils. The obtained results (Table 1), indicate that soil leachates are characterized by trace metal concentrations below the MCL reported for groundwater by Italian regulations, except Sb and Fe.

Trace element data on Pontecosi lake sediments are reported in Table 2. Sediments are characterized by relatively high Mn content (between 440 and 1090 mg/kg), as observed in basalt-hosted deposits in Apennine ophiolites, and Fe (between 18.8 and 31.2 g/kg); the lack of Fe vs. Mn correlation (not shown) likely reflects the complex cycling of these redox-sensitive trace metals in lacustrine environment [33]. Vanadium, Cr and Ni concentrations are in the range from 23 to 58 mg/kg, 55 to 111 mg/kg and 55 to 96 mg/kg, respectively. Vanadium vs. Cr and Ni vs. Cr show a positive covariance and plot along single, well-defined regression lines (not shown; R^2^ = 0.82 and R^2^ = 0.86, respectively) with no distinction between subaerial and lake floor deposits. This suggests that hydraulic processes and grain-size do not significantly influence the sediment composition. Copper, Zn and Pb concentration ranges from 18 to 51 mg/kg, 48 to 59 mg/kg and 6.7 to 20 mg/kg, respectively. Despite some scatter, Pb vs. Zn and Pb vs. Cu correlate linearly (not shown; R^2^ = 0.89 and R^2^ = 0.73, respectively), a feature associated to the (Fe)–Cu–Zn–Pb sulfides that characterize ophiolites of the Ligurian Units in northern Apennine, currently exposed north of Castelnuovo di Garfagnana at the bottom of the Serchio Valley [34] (Figure 1).

The Co/Ni ratio is quite constant (Co/Ni = 0.20 ± 0.01), also resembling what reported for pyrites in serpentinite-hosted deposits of northern Apennine ophiolites. Arsenic ranges between 2.9 and 5.5 mg/kg, likely reflecting a source from pyrite in sulfide ores. These observations indicate that trace metals are supplied to sediments by ophiolite-rich sources. Strontium ranges from 41 to 350 mg/kg, reflecting inputs from the carbonate rocks. Potentially toxic elements in Pontecosi lake sediments are all below the MCL for soils by Italian regulations (see Table 1 and Table 2), and can be used to establish reliable natural trace elements (including PTE) concentration in downstream Serchio River sediments and related soils.

### 4.2. Groundwater

**Figure 4:**
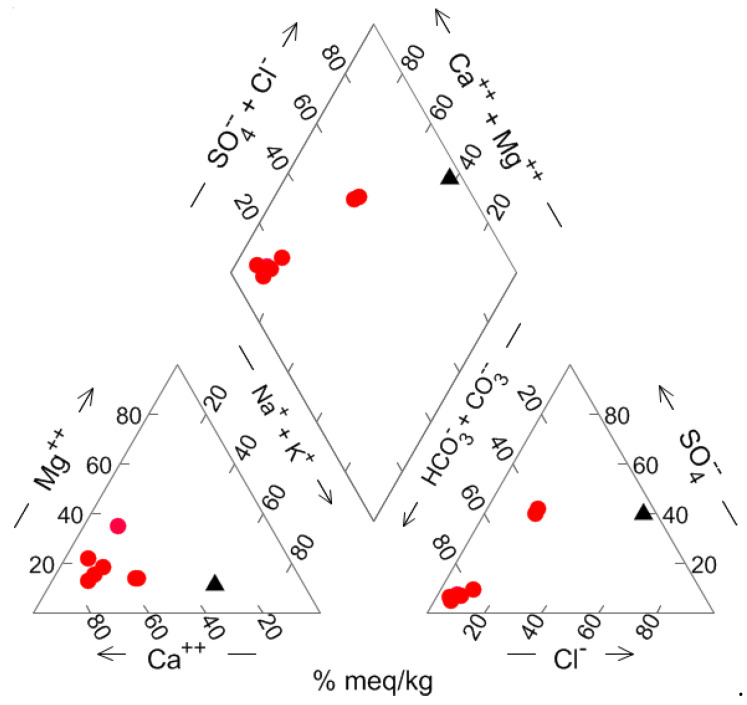
Piper diagram for groundwater samples. Red circles: FB_1W to FB_6W wells and piezometer; black filled triangle: deep water (see text).

The physico-chemical parameters and major element chemistry measured on groundwater are given in Table 3; trace elements analyses are reported in Table 4. The major ions chemistry is graphically shown in the Piper diagram of Figure 4.

The diamond-shaped field in the Piper diagram classifies groundwater as belonging to the Ca-HCO_3_ type; it has however to be noted that the water samples FB_6Wa,b are shifted toward SO_4_^2−^, Cl^−^ and Na^+^ enrichments, a feature that characterizes the end-member thermal waters outflowing in the area and interpreted as reflecting a deep circulation in Triassic carbonate-evaporite aquifers [35].

Trace element contaminants in groundwater have concentration below the MCL reported by Italian regulations, except Mn that exceed the maximum concentration value of 50 µg/L in FB_3W and FB_6W.

### 4.3. Risk Analysis

Health risk assessment was calculated for Cu, Zn and Cd (Table 5), exceeding the MCL for soil (Table 1) imposed by Italian regulations. The highest measured concentrations were precautionary used (Table 1) due to the small sample size [36]. Sample FB_14S, interpreted as a primary source of contamination, was not included in calculations.

The obtained HI_cum_ (0.956, Table 5) was only slightly lower than the acceptance threshold. The calculated individual and cumulative soil screening levels (SSLs,) are given in Table 6. It can be observed that the obtained screening values are very close to the maximum concentrations measured in soil.

## 5. Discussion

Potentially toxic elements data indicate that Fornaci di Barga soils are variably contaminated by Cu, Zn, Cd, exceeding the thresholds for residential use. It must be noted that these elements in sample FB_5S, a station in a rural setting, do not exceed the MCL for soil, suggesting that contamination is confined to the urbanized environment, in particular in proximity of the metallurgical plant. Soil in station FB_14S, near the industry (Figure 2a), deviates towards the highest level of pollution, including Ni, Sb, Pb, As and Hg in addition to Cu, Zn and Cd. These data suggest that contaminants enter the environment from a confined space, possibly representing the legacy of waste storage. The extent of this polluted area suspected to be hazardous must be investigated and clean-up procedures for remediation should be planned.

**Figure 5:**
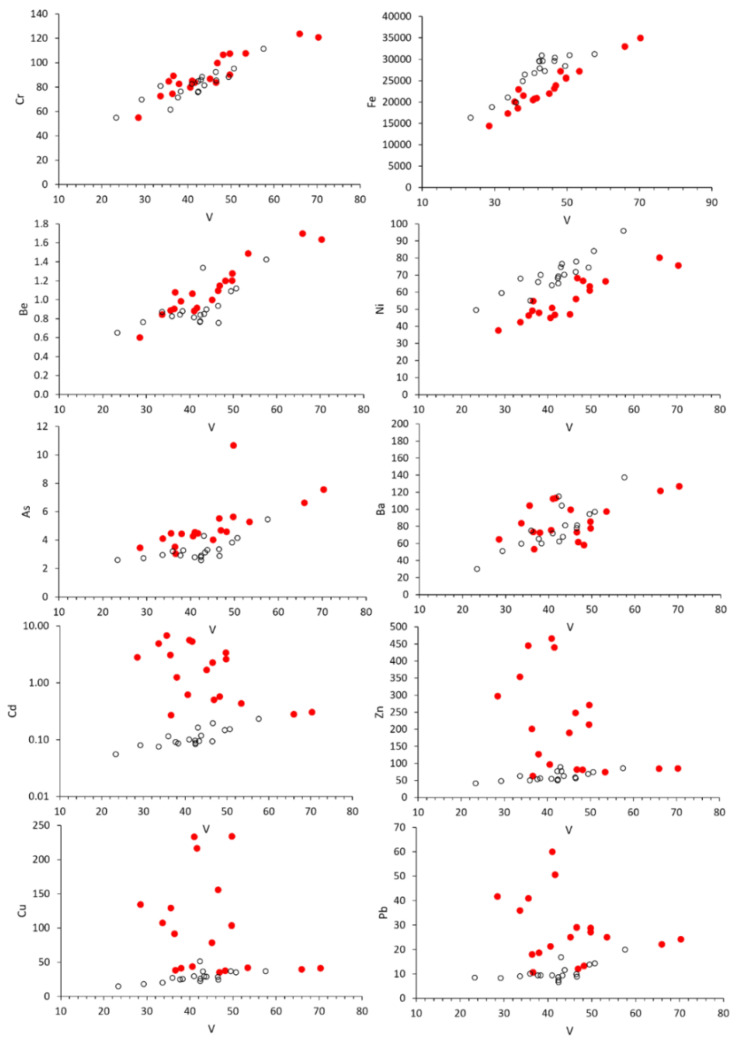
Cr, Fe, Be, Ba, Ni, As, Cu, Zn, Cd and Pb concentrations (mg/kg) vs. V (mg/kg) in Fornaci di Barga soils (filled circles) and Pontecosi lake sediments (open circles).

In order to characterize natural background concentrations of PTE in soils, the Pontecosi lake sediments unaffected by pollution have been used as reference conditions. In particular, vanadium has been intended as an indicator for ophiolitic sources. It is observed that Fornaci di Barga soils and Pontecosi lake sediments have similar patterns for Cr, Fe, Be, Ba, Ni, As vs. V (Figure 5), suggesting common lithogenic sources. On the contrary, deviations towards higher concentrations compared with natural values are observed for Cu, Zn, Cd and Pb vs. V (Figure 5), even if Pb does not exceed the maximum concentration level for residential soils.

The observed trends confirm the input of Cu, Zn and Cd from anthropogenic sources. To assess the contribution of anthropogenic deposition on surface soil, enrichment factor (EF) for Cu, Zn and Cd, exceeding the maximum concentration level, has been calculated using iron as a reference element and the relationship:

EF=(MeFe)sample(MeFe)UCC

where *Me* represent the metal concentration and *UCC* the upper continental crust [37]. The results indicate an extremely high enrichment for Cd (EF > 40) and moderate to significant enrichments for Zn and Cu (2 < EF < 5 and 5 < EF < 10, respectively). Soil pollution has been also evaluated by using the geo-accumulation index (I_geo_) calculated by using the Müller relationship [38] given by:

$$I_{geo}=log_{2}\left(\frac{Cn}{1.5Bn}\right)$$

where *Cn* represents the element concentration in soil and Bn the geogenic background level for individual element taken as the concentration measured in the Pontecosi lake sediments. The obtained results highlight that most soil samples belong to Class 6 (*I_geo_* > 5, extremely contaminated soil) for Cu, Zn, Cd. Similar results are obtained by using background concentration values for sediments representative of the Earth’s crust [37,39].

The calculated SSL concentration values, that are deemed protective of receptors that come into contact with soil, reveal that the highest measured Cu, Zn and Cd concentration in Fornaci di Barga soil (FB_3S, FB_2S and FB_13S stations, respectively) should be considered of potential toxicological concern. Risk assessment indicates that the major threat for health is associated to Cd through soil ingestion pathway by children. Dermal contact pathway for Cu, Zn and Cd does not rise potential adverse health effects.

The local, long-lasting metallurgical activity might be responsible for Cu, Zn, Pb and Cd emission; in particular, gaseous Cd volatilizes and condense forming particulate [40,41]. Road traffic also represents an emission source for trace metal loading on soil [42,43]; in particular, Cu is released during vehicle brake abrasion, Zn by automotive tire wear, Pb mostly represents the legacy of burned leaded gasoline [44]. It has to be noted that the highest concentration of PTE has not been measured in roadside environments, suggesting that the major contribution is from industrial activity. However, additional studies are necessary to unequivocally identify the main anthropogenic sources of pollutant emission in the area.

**Figure 6:**
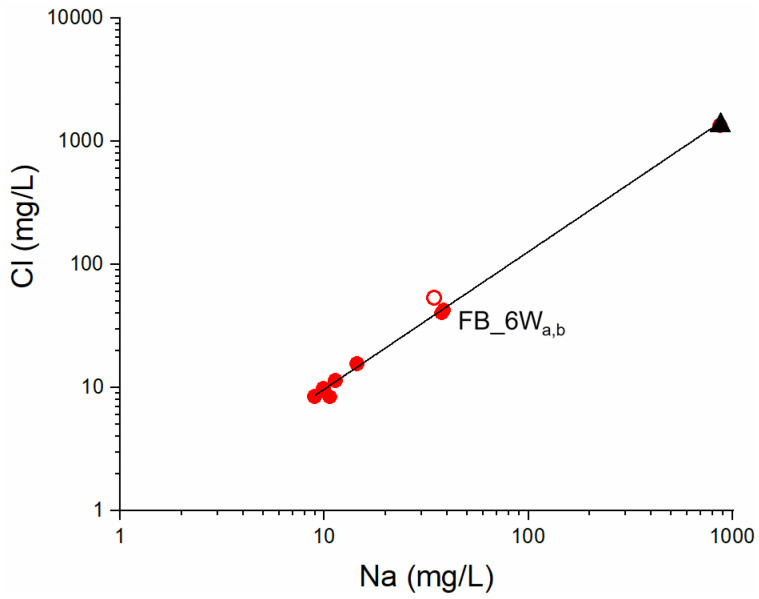
Na (mg/L) vs. Cl (mg/L) diagram with the linear fitting (black line). Red filled circles: groundwater from FB_1W to FB_6W; red open circle: modelled FB_6W water (see text); black filled triangle: deep water (see text).

Soil profiles show that Cu, Zn and Cd concentration gradually decreases with increasing depth, confirming that the anthropogenic contribution correlates with local air active emission sources. Furthermore, experiments indicate that Cu, Zn and Cd concentrations in soil leachates (Table 1) were significantly higher than those measured in groundwater samples (Table 4), suggesting that soils have the potential to release contaminants according to the principle that elements of accumulated anthropogenic nature have the tendency to be mobile in soil when compared with those originated from lithogenic sources [45]. Once below the water table, contaminants may be subject to dispersion and diffusion, with different flowpaths and velocities [46], possibly extending groundwater contamination. The geochemical data on samples FB_6Wa,b show a relatively high Li, Ba, Cl, Na and SO_4_ concentration (Table 3 and Table 4), interpreted as the evidence of a deep-water component in evaporitic-carbonatic hydrogeological structures rising to the surface through fault systems [35]. The linear correlation in the binary Na vs. Cl diagram (Figure 6) highlights the contribution of such deep component to the shallower groundwater in alluvial deposits. In particular, the FB_6W water chemistry may be simulated using the PHREEQC Code [47] by 4% mixing of the deep water (represented by Pieve di Fosciana outflows [35] (Figure 6) with modelled Ca-HCO_3_ water in equilibrium with calcite and undersaturated with dolomite (saturation index = −0.5) in a system closed to CO_2_. These observations suggest that mixing-induced transport might occur in cases when soil leachates reach saturated strata, and contaminants might in this case travel over variable distances from the discharge point. Groundwater monitoring should hence be carried out regularly.

## 6. Conclusions

The results obtained in this study indicate that Cu, Zn and Cd, likely originated by deposition of airborne particulate matter released during the historical and long-lasting metallurgical activity that characterized the Fornaci di Barga area, represent the main contaminants in surface soil.. Even low-level environmental exposure to cadmium is a risk factor for lung cancer [48,49] and may potentiate the effects diabetes on the kidney and the chronic kidney disease [50,51,52]. Risk assessment indicates that cadmium may contribute to adverse health outcomes for children by direct soil ingestion exposure routes. The calculated general soil screening level closely approaches the highest measured contaminant concentration in soil. Urban groundwater does not show evidence of trace element pollution, suggesting that the migration of contaminants from soil to subsurface is a slow process. However, the hydrologic interconnection between shallow and deep aquifers through fault zones requires a network monitoring.

These results represent the first evidence of the links between space clusters of diseases, soil contamination and risk assessment in the densely populated area of the Middle Serchio River Valley, and provide the scientific insights to policy makers and stakeholders for future-oriented solutions.

## Figures and Tables

**Table 1:** Trace element concentration in Fornaci di Barga soils (mg/kg) and in soil leachates (µg/L) from FB_13S and FB_14S samples) together with the maximum concentration level (MCL, mg/kg) imposed by Italian regulations (Legislative Decree No. 152/2006 approving the Code on the Environment). Values in bold exceed the MCL for residential soil use or groundwater in case of leachates.

Sample	Depth	Li	Be	Mn	Co	Ni	Cu	Zn	Sr	Mo	Ag	Sn	Cd	Sb	Ba	Tl	Pb	Th	U	V	Cr	Fe	As	Hg
FB_1Sa	0–10	43	1.70	892	16.1	80	39	84	41	0.72	0.12	2.3	0.28	0.27	121	0.33	22.1	7.1	1.42	66	123	32,900	6.6	0.05
FB_1Sb	50–60	41	1.63	1120	16.3	75	41	84	43	0.70	0.16	2.5	0.30	0.42	127	0.36	24.2	5.8	1.22	70	121	34,900	7.5	0.07
FB_2Sa	0–10	22	0.91	707	8.0	47	**216**	**439**	92	<0.5	0.16	4.1	**5.3**	0.57	113	0.25	51	5.1	1.00	42	84	20,900	4.5	0.31
FB_2Sb	20–30	20	0.88	719	8.0	51	**233**	**465**	94	<0.5	0.15	4.9	**5.6**	0.84	112	0.25	60	4.4	1.00	41	85	20,700	4.5	0.34
FB_3S	0–10	28	1.27	951	15.2	63	**234**	**270**	26	<0.5	0.08	2.2	**2.6**	0.55	77	0.28	27	5.0	0.9	50	90	25,700	10.6	0.10
FB_4S	0–10	22	1.06	519	8.2	45	43	96	23	<0.5	0.07	2.1	0.61	0.42	75	0.22	21	4.8	1.00	41	80	20,400	4.3	0.07
FB_5S	0–10	31	1.49	754	11.5	66	42	74	31	<0.5	0.05	2.0	0.43	0.42	97	0.29	25	6.1	1.27	53	107	27,200	5.3	0.06
FB_6S	0–10	31	1.20	768	10.1	61	103	**213**	29	<0.5	0.08	2.3	**3.3**	0.51	85	0.27	29	5.6	1.38	50	107	25,500	5.6	0.09
FB_7S	0–10	32	1.20	506	11.2	66	37	80	31	<0.5	0.05	1.50	0.57	0.22	58	0.19	13.2	5.4	1.06	48	106	27,100	4.6	0.04
FB_8S	0–10	23	1.07	377	9.4	55	38	62	26	<0.5	0.05	1.39	0.27	0.20	53	0.15	10.6	5.2	1.06	37	89	22,900	3.0	0.06
FB_9S	0–10	19.9	0.90	427	8.1	49	91	**200**	79	0.22	0.06	1.81	**3.1**	0.29	73	0.20	17.9	4.4	0.92	36	74	18,500	3.5	0.04
FB10S	0–10	27	1.15	607	10.9	68	35	81	33	0.22	0.03	1.43	0.50	0.23	61	0.19	12.0	6.3	1.08	47	100	23,800	4.7	0.05
FB_11S	0–10	26	1.09	1017	12.0	56	**155**	**248**	25.6	0.36	0.09	2.3	**2.2**	0.51	73	0.29	29	4.7	0.86	47	84	23,100	5.5	0.16
FB_12Sa	35–50	24	0.99	701	9.7	47	78	**189**	56	0.75	0.13	2.3	1.67	0.34	99	0.33	25	4.8	1.09	45	87	21,900	4.0	0.11
FB_12Sb	50–60	14.4	0.60	636	6.3	37	**134**	**296**	78	0.28	0.13	2.9	**2.8**	0.5	65	0.21	42	3.0	0.67	28.6	55	14,300	3.4	0.38
FB_13Sa	20–30	19.5	0.88	455	7.6	46	**129**	**445**	23.5	0.70	0.26	4.5	**6.7**	0.61	104	0.23	41	5.6	1.26	36	84	20,000	4.5	0.23
FB_13Sb	40–50	18.2	0.84	381	7.0	42	107	**353**	20.1	0.42	0.18	3.7	**4.8**	0.49	83	0.22	36	5.1	1.27	34	72	17,300	4.1	0.22
FB_13Sc	60–70	23	0.98	401	8.4	48	41	126	17.9	0.36	0.11	2.0	1.20	0.36	72	0.24	18.6	6.6	1.36	38	82	21,400	4.4	0.16
FB_14S	15–22	32	1.24	1530	16.5	**265**	**5280**	**27,400**	46	1.37	8.5	22.1	**15.8**	**11.7**	169	0.23	**221**	5.3	1.29	55	146	34,800	**255**	**2.2**
MCL			2		20	120	120	150					2	10		1	100			90	150		20	1
FB13a_leach_		<4	<0.2	9.0	<0.2	<4	58	153	<36	<5	<0.4	<2	1.41	1.15	<14	<0.15	<4	0.16		<2.5	<2.5	**479**	1.10	
FB14_leach_		<4	<0.2	<4	<0.2	<4	47	178	40	<5	<0.4	<2	1.20	**6.9**	183	<0.15	<4	<0.06		<2.5	<2.5	46	0.94	
MCL			4	50	50	20	1000	3000					5	5		2	10	<0.06			50	200	10	

**Table 2:** Trace elements on Pontecosi lake sediments (mg/kg).

Sample	Li	Be	Mn	Co	Ni	Cu	Zn	Sr	Mo	Ag	Sn	Cd	Sb	Ba	Tl	Pb	Th	U	V	Cr	Fe	As	Hg
PC1	25	0.77	1090	13.4	69	22	50	350	<0.4	0.08	0.19	0.08	<0.07	253	0.08	6.7	3.5	0.48	42	85	29,500	2.6	<0.07
PC2	29	0.76	1050	13.6	68	51	77	162	<0.4	0.08	0.33	0.10	<0.07	115	0.08	8.7	3.4	0.49	42	76	29,600	2.8	<0.07
PC3	28	0.84	710	12.3	66	25	53	131	<0.4	0.06	0.21	0.09	<0.07	65	0.10	9.5	4.0	0.58	38	71	24,900	2.9	<0.07
PC4	27	0.76	930	14.4	78	24	56	173	<0.4	0.08	0.19	0.19	<0.07	81	0.08	8.9	3.5	0.48	47	85	30,400	2.9	<0.07
PC5	27	0.81	630	13.0	64	30	55	129	<0.4	0.08	0.17	0.10	<0.07	72	0.11	9.4	4.3	0.62	41	83	26,800	2.8	<0.07
PC6	29	0.94	710	13.3	72	28	59	131	<0.4	0.06	0.27	0.09	<0.07	78	0.12	9.9	5.7	0.85	47	92	29,600	3.4	<0.07
PC7	30	0.90	670	13.9	70	29	63	125	<0.4	0.07	0.19	0.12	<0.07	81	0.12	11.6	4.8	0.80	44	81	27,300	3.3	<0.07
PC8	36	1.12	810	16.7	84	35	74	149	<0.4	0.08	0.24	0.15	<0.07	97	0.15	14.4	6.4	1.00	51	95	30,900	4.2	<0.07
PC9	30	0.88	720	12.9	70	26	56	123	<0.4	0.12	0.21	0.09	<0.07	60	0.09	9.4	4.5	0.64	38	76	26,400	3.3	<0.07
PC10	28	0.83	600	10.6	55	27	50	115	<0.4	0.08	0.15	0.11	<0.07	75	0.13	10.1	3.6	0.62	36	62	19,900	3.2	<0.07
PC11	35	1.09	820	14.8	74	37	69	166	<0.4	0.09	0.37	0.15	<0.07	94	0.16	13.9	5.1	0.86	49	88	28,400	3.8	<0.07
PC12	42	1.34	650	16.1	75	37	89	100	<0.4	0.07	0.21	0.16	<0.07	104	0.21	16.9	5.6	0.80	43	86	30,900	4.3	<0.07
PC13	25	0.76	440	10.1	59	18	48	41	<0.4	<0.03	0.24	0.08	<0.07	51	0.09	8.3	5.1	0.82	29	70	18,800	2.7	<0.07
PC14	41	1.43	830	17.0	96	37	86	88	<0.4	0.06	0.49	0.23	<0.07	137	0.23	20.0	7.0	1.48	58	111	31,200	5.5	<0.07
PC15	27	0.84	730	12.6	65	26	53	136	<0.4	0.06	0.19	0.09	<0.07	62	0.09	7.5	4.5	0.55	42	76	27,900	2.9	<0.07

**Table 3:** The physico-chemical parameters and major ions chemistry (mg/L) measured on groundwater.

Sample	Depth	T_w_ (°C)	T_air_ (°C)	DO (mg/L)	EC (mS)	pH	Na^+^	K^+^	Mg^2+^	Ca^2+^	F^−^	Cl^−^	NO_3_^−^	SO_4_^2−^	HCO_3_^−^
FB_1W	50	14.0	30.2	3.5	293	7.3	11	1.2	6.4	60	<0.2	8.4	5.8	9.1	210
FB_2W	8	16.6	30.2	2.4	391	6.6	15	1.1	9.6	58	0.3	16	32	17	183
FB_3W	7	16.4	30.2	2.0	296	6.9	9.9	1.0	16	40	0.3	9.7	3.2	14	207
FB_4W	18	16.2	30.2	1.0	432	7.3	11	1.0	16	84	<0.2	11	1.5	18	332
FB_5W	21	14.9	30.2	3.8	266	6.9	9.0	1.0	5.6	43	<0.2	8.4	7.5	8.8	143
FB_6Wa	2	17.0	15.4	6.1	520	7.6	39	3.5	10	68	n.d.	42	n.d.	132	159

n.d.: not determined.

**Table 4:** Trace elements in groundwater (µg/L) together with the maximum concentration level (MCL, µg/L) imposed by Italian regulations (Legislative Decree No. 152/2006 approving the Code on the Environment). Values in bold exceed the concentration threshold for groundwater.

Samples	Li	Be	Mn	Co	Ni	Cu	Zn	Sr	Mo	Ag	Sn	Cd	Sb	Ba	Tl	Pb	Th	U	V	Cr	Fe	As
FB_1W	1.46	<0.01	5.5	0.12	3.8	2.0	<7	166	0.20	<0.02	<0.1	0.02	0.16	39	0.01	<0.2	0.006	0.17	0.23	0.23	53	0.14
FB_2W	0.26	<0.01	1.06	0.18	3.7	2.4	9	156	<0.2	<0.02	<0.1	0.02	0.04	28	<0.006	0.34	<0.006	0.11	0.58	0.55	50	0.10
FB_3W	4.1	<0.01	**306**	0.11	2.6	2.2	14	130	<0.2	<0.02	<0.1	0.03	0.04	4.7	0.01	<0.2	<0.006	0.19	0.21	<0.1	60	0.10
FB_4W	2.7	<0.01	10.6	0.18	4.8	<1	41	189	<0.2	<0.02	<0.1	0.01	0.04	13.2	<0.006	<0.2	<0.006	0.27	<0.1	0.11	123	0.09
FB_5W	1.15	<0.01	0.50	0.08	2.4	3.4	77	131	<0.2	<0.02	<0.1	0.02	0.05	22	<0.006	<0.2	<0.006	0.14	0.20	0.21	34	0.05
FB_6Wa	35	<0.01	**199**	0.20	4.4	2.4	<7	570	1.59	0.03	0.11	0.04	0.18	48	0.14	<0.18	<0.006	0.33	0.48	<0.12	7.0	0.39
FB_6Wb	35	<0.01	**155**	0.35	4.4	1.10	<7	552	1.76	<0.02	<0.1	0.03	0.20	52	0.01	<0.18	<0.006	0.39	0.51	<0.12	9.0	0.47
MCL		4	**50**	50	20	1000	3000					5	5		2	10				50	200	10

**Table 5:** Summary of Hazard Quotient (HQ) for non-carcinogenic risk calculated for each contaminant and for direct exposure routes, for children and adults receptor (in brackets values for adult when different). The individual (HI_i_) and cumulative (HI_cum_) Hazard Index are also reported.

HQ	Soil Ingestion	Dermal Contact	HI_i_
Cadmium RfD_oral_ = 1 × 10^−4^ mg/kg/day	0.857 (9.18 × 10^−2^)	2.40 × 10^−3^ (3.66 × 10^−4^)	0.859 (9.22 × 10^−2^)
Copper RfD_oral_= 4 × 10^−2^ mg/kg/day	7.48 × 10^−2^ (8.01 × 10^−3^)	2.09 × 10^−3^ (3.20 × 10^−4^)	7.69 × 10^−2^ (8.33 × 10^−3^)
Zinc RfD_oral_ = 3 × 10^−1^ mg/kg/day	1.98 × 10^−2^ (2.12 × 10^−3^)	5.55 × 10^−4^ (8.47 × 10^−5^)	2.04 × 10^−2^ (2.21 × 10^−3^)
Sum of individual hazard quotients for each route considered	0.952 (0.102)	5.05 × 10^−3^ (7.71 × 10^−4^)	HI_cum_ (all contaminants) 0.956 (0.103)

**Table 6:** Summary of individual SSLs for Cadmium, Copper and Zinc respectively calculated for direct exposure routes, for children and adults receptor (in brackets values for adult). * Calculated according to USEPA [32]; ** SSLs recalculated taking into account the simultaneous presence of all contaminants (applying a corrective factor, *f*).

Individual SSL	Soil Ingestion	Dermal Contact	Sum of Direct Exposures *	*f*	Cumulative SSL **
Cadmium C_max_ = 6.7 mg/kg	7.82 (7.30 × 10^1^)	2.79 × 10^3^ (1.83 × 10^4^)	7.80 (7.27 × 10^1^)	1.11	7.03 (6.55 × 10^1^)
Copper C_max_ = 234 mg/kg	3,13 × 10^3^ (2.92 × 10^4^)	1,12 × 10^5^ (7.32 × 10^5^)	3.04 × 10^3^ (2.81 × 10^4^)	13	2.34 × 10^2^ (2.16 × 10^3^)
Zinc C_max_ = 465 mg/kg	2.35 × 10^4^ (2.19 × 10^5^)	8.38 × 10^5^ (>10^6^)	2.28 × 10^4^ (2.11 × 10^5^)	49	4.66 × 10^2^ (4.30 × 10^3^)

## Data Availability

Not applicable.
